# Local recurrence and assessment of sentinel lymph node biopsy in deep soft tissue leiomyosarcoma of the extremities

**DOI:** 10.1186/2045-3329-1-7

**Published:** 2011-08-01

**Authors:** Michael J Lamyman, Henk P Giele, Paul Critchley, Duncan Whitwell, Max Gibbons, Nicholas A Athanasou

**Affiliations:** 1 Nuffield Department of Orthopaedics, Rheumatology and Musculoskeltal, Sciences, University of Oxford, Department of Pathology, Nuffield Orthopaedic Centre, Oxford, OX3 7LD, UK

## Abstract

**Background:**

Leiomyosarcoma of deep soft tissues of the extremities is a rare malignant tumour treated primarily by surgery. The incidence of local recurrence and lymph node metastasis is uncertain and it is not known whether a sentinel lymph node biopsy is indicated in these tumours.

**Methods:**

A retrospective review of patients treated for extremity deep soft tissue leiomyosarcoma at our institution over a 10-year period was conducted. Patients developing local recurrence or lymph node metastasis were identified. The presence or absence of lymphatics in the primary tumours was assessed by immunohistochemical expression of LYVE-1 and podoplanin.

**Results:**

27 patients (mean age 62 years) were included in the study. 15 were female and 12 male. Lymph node metastasis was seen in only two cases (7%); intratumoural lymphatics were identified in the primary tumours of both these cases. Local recurrence occurred in 25.9% of cases despite complete excision and post-operative radiotherapy; the mean time to recurrence was 10.1 months.

**Conclusion:**

On the basis of this study, we do not advocate sentinel lymph node biopsy in this group of patients except in those cases in which intratumoural lymphatics can be demonstrated. Close follow up is important especially for high grade leiomyosarcomas, particularly in the first year, as these tumours have a high incidence of local recurrence.

## Introduction

Leiomyosarcoma of soft tissues is a malignant tumour composed of tumour cells that exhibit smooth muscle differentiation. Leiomyosarcomas are generally thought to account for 5-10% of soft tissue sarcomas [1-3]. These tumours arise most commonly in the retroperitoneum but can develop in any location; in one study of 75 soft tissue leiomyosarcomas, 33% were noted to arise in extremity soft tissues\The behaviour of leiomyosarcoma of extremity deep soft tissues has not been studied independently of those arising in other locations.

Regional lymph node metastasis in patients with soft tissue sarcomas is an infrequent event occurring in 2.6 - 5% of all patients [4-6]. Sentinel lymph node biopsy (SLNB) has been employed for staging of soft tissue sarcomas, particularly epithelial sarcoma [7-9]. The incidence of lymph node metastasis in extremity leiomyosarcomas is clearly important with regard to whether SLNB should be carried out for this tumour. In previous retrospective reviews of the literature, pooling data from published reports on regional lymph node involvement, Weingrad and Rosenberg [10] and Mazeron and Suit [11] found the incidence in leiomyosarcoma was 10.6% and 4% respectively; in the prospective study of Fong et al [5], the incidence was reported to be 2.7%. These studies, however, did not distinguish leiomyosarcoma of extremity deep soft tissues from those arising in other locations; this is an important factor as leiomyosarcoma occurs more commonly in the retroperitoneum, mesentery, abdominal and pelvic viscera than in extremity soft tissues and lymph node metastasis from sarcomas of visceral origin occurs less commonly than from sarcomas arising in extremity soft tissues [5]. The recurrence rate following excision of deep soft tissue extremity leiomyosarcomas is also unknown; this has not been assessed independently of recurrence of superficial (cutaneous) leiomyosarcomas, which have a favourable prognosis, or of retroperitoneal tumours, which have a poor prognosis.

The aim of this study has been to determine the recurrence rate and incidence of lymph node metastasis of deep soft tissue leiomyosarcomas of the extremities. As the presence of lymphatic vessels has been noted in malignant soft tissue tumours that metastasise to lymph nodes [12], we determined whether immunohistochemical identification of lymphatics in the primary tumour could provide a guide as to whether lymph node metastasis of extremity leiomyosarcoma occurred and thus, whether a SLNB might be indicated in such cases.

## Patients and methods

A search of the pathology database detected all patients with a histological diagnosis of deep soft tissue leiomyosarcoma over a 10 year period, between 1998 and 2008. Only patients diagnosed and treated at the Nuffield Orthopaedic Centre with leiomyosarcoma of the extremities were entered into the study. Patients with superficial cutaneous soft tissue leiomyosarcomas or gynaecological, retroperitoneal, intra-abdominal or intrathoracic primary tumours were excluded. A case notes review was performed.

Local recurrence and lymph node metastasis was considered to have occurred only if proven though open biopsy. The histological diagnosis of leiomyosarcoma was based on morphological and immunohistochemical criteria detailed in the WHO classification of soft tissue tumours [1]. Immunohistochemical expression of at least two smooth muscle antigens (smooth muscle actin, desmin, h-caldesmon) was seen in all cases. Identification of lymphatics was carried out using anti-Lyve -1 and anti-podoplanin antibodies as previously described [12].

## Results

**Figure 1:**
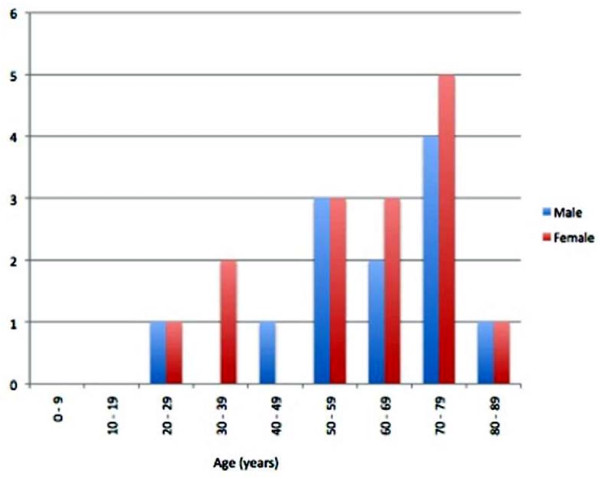
**Age and sex distribution of cases**.

**Figure 2:**
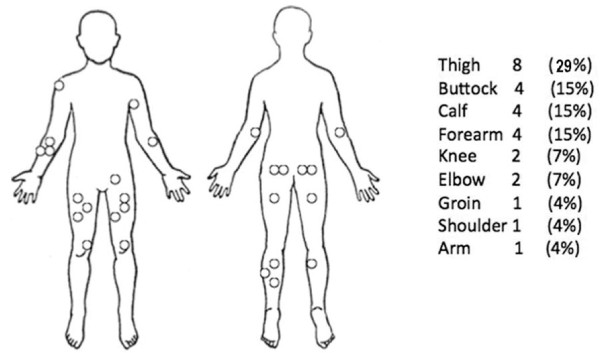
**Sites of primary tumour with number and approximate percentage of cases**.

35 patients were identified as eligible for entry into the study. Five patients had to be excluded as either the case notes could not be found or were incomplete. Two patients were excluded because they died following their biopsy but before definitive surgery, and one patient was excluded because metastatic disease was found on presentation. The case notes of the remaining 27 patients were reviewed. Fifteen were female and twelve male (Figure 1). The mean age at presentation was 62 years. The mean follow up was 19.9 months, median 15 months (range 4 to 59 months). The sites of the primary tumour are shown in Figure 2. The size, grade and stage of the tumours are shown in Table 1. In all cases, local excision of the tumours was performed aiming for complete clearance with as wide a margin as possible. 21 of the patients (78%) received adjuvant radiotherapy following primary excision. Details of patients developing local recurrence are shown in Table 2 and of those developing lymph node metastasis in Table 3. Lymph node metastasis occurred in two patients (7%).

**Table 1:** Size, Grade and Stage of the primary leiomyosarcoma

Case	Size in maximum Diameter (cm)	Grade	MSTS Stage
1	18	2	2b
2	8	3	2b
3	5	3	2b
4	Unknown	2	2b
5	7.5	1	lb
6	4.5	2	lb
7	10	3	2b
S	7	3	2b
9	4	2	2b
10	8	3	2b
11	13	3	2b
12	14	3	2b
13	2	2	2b
14	3.8	1	lb
15	12	2	2b
16	25	2	2b
17	12	2	2b
18	3.5	2	2b
19	8.5	3	2b
20	10	3	2b
21	16	3	2b
22	9	1	lb
23	13	1	lb
24	9	3	3
25	Unknown	2	2b
26	10	3	2b
27	8	3	2b

MSTS - Musculoskeletal Tumour Society

**Table 2:** Patients with local recurrence following primary excision

Age	Sex	Site	Max Diam eter (cm)	Grade	MSTS Stage	Time to first recurrenc e (Months)	Number of recurrences	Margins	Adjuvant Radio therapy
55	F	Arm	5	3	2b	3	2	Clear	Yes
77	F	Calf	Unknown	2	2b	24	2	Clear	Yes
77	M	Buttock	8	3	2b	3	4	Clear	Yes
67	F	Thigh	13	3	2b	9	2	Clear	Yes
67	M	Buttock	12	2	2b	4	2	Clear	Yes
55	F	Calf	9	3	2b	5	1	Marginal	Yes
76	M	Forearm	unknown	2	2b	19	1	Clear	Yes

**Table 3:** Patients with lymph node metastasis

Age	Sex	Site	Max Diameter (cm)	Grade	MSTS Stage	Margins	Aduvant Radiotherapy	Time to detection of lymph node metastasis (months)
79	F	Thigh	8.5	3	2b	Clear	Yes	21
53	M	Thigh	8	3	2b	Clear	No	3

**Figure 3:**
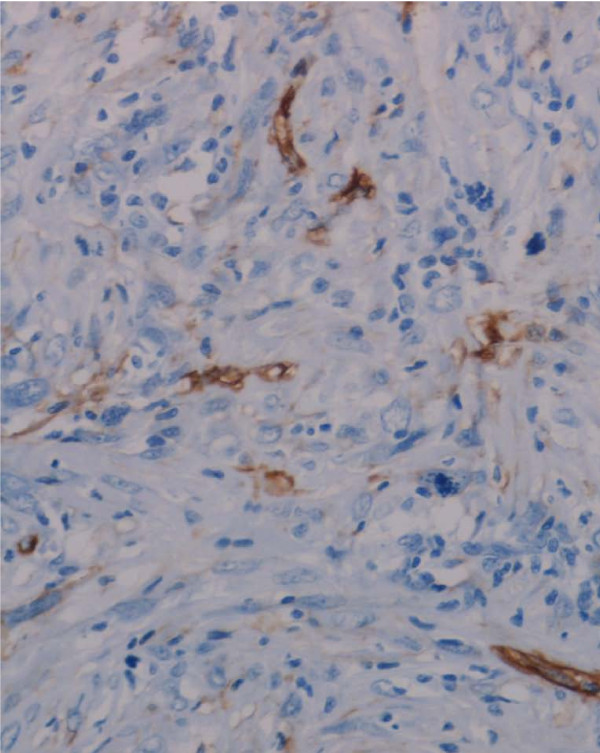
**Intratumoural lymphatic vessels in a primary leiomyosarcoma, showing podoplanin expression by lymphatic endothelial cells**.

A review of the pathology of the primary tumour in these two cases showed that both tumours contained intratumoural lymphatics, as assessed by endothelial cell expression of the lymphatic markers, podoplanin and LYVE-1. (Figure 3) The remaining tumours, which did not metastasise to lymph nodes, were negative for lymphatic markers. In one patient the nodal recurrence was extensive, encasing femoral vessels and was not resectable. In the second patient an inguinal and iliac lymph node dissection was performed. In this patient the lymph node metastasis occurred early, before radiotherapy was instituted.

Local recurrence occurred in seven patients (25.9%). The mean time from surgical excision to recurrence was 10.1 months (range 3-24 months). There was no incidence of local recurrence or lymph node metastasis in patients with low grade leiomyosarcoma. Post-operative radiotherapy was received by all patients who subsequently presented with local recurrence. In six of these seven patients, the tumour had been excised with a clear margin. In one patient the excision was described as marginal. In all but one case the recurrence was treated by further surgical resection.

## Discussion

The role of SLNB in the management of soft tissue sarcoma has yet to be defined [8,9,13]. In our institution it is current practice to undertake SLNB in patients with epithelioid sarcoma given the relatively high rate of lymph node metastasis in these tumours. Previous studies have reported that the incidence of lymph node metastasis in such tumours is between 16.7 and 80% [5,10,11]. A positive SLNB in these cases is followed by a formal lymph node dissection. A number of soft tissue sarcomas, such as rhabdomyosarcoma, clear cell sarcoma and synovial sarcoma, have also been shown to have a propensity for regional lymph node metastasis and some observers have suggested that SLNB may be of prognostic benefit in these tumours [9]. Previous estimates of the incidence of lymph node metastasis in all patients with leiomyosarcoma have been between 2.7 and 10.6%. ^[510H] ^These studies examined the metastatic rate of leiomyosarcomas arising at several different sites collectively and not just that of leiomyosarcomas of deep soft tissues of the extremities. In the present study we found that the rate of lymph node metastasis in extremity deep soft tissue leiomyosarcomas to be 7%.

In patients with intermediate thickness melanoma, SLNB has become widely accepted as a minimally invasive method of staging the regional lymph nodes [14,15]. When SLNB is performed in these patients, 20% will be found to have micrometastasis. However when SLNB is performed in thin melanomas, with a Breslow thickness less than I mm, the micrometastasis rate falls to 5% [16]. Current AJCC guidelines do not recommend routine use of SLNB in this group [17,18], and on this basis the comparable rate of lymph node metastasis in deep soft tissue leiomyosarcomas would not appear to justify the extra morbidity (eg extra operating time, potential wound problems) associated with undertaking SLNB.

Recent work at our institution has shown that soft tissue sarcomas with a high propensity to metastasise to lymph nodes contain intratumoural lymphatics [12]; intratumoural lymphatics were found to be present in all epithelioid sarcomas and a number of other sarcomas including leiomyosarcoma. Lymph node metastasis has been reported in up to 80% of epithelioid sarcomas [5,10,11]. The lower incidence of lymph node metastasis in leiomyosarcomas may reflect the fact that intratumoural lymphatics are found less commonly in these tumours. It is none the less significant that in our study the two leiomyoarcomas which did metastasise to regional lymph nodes both contained intratumoural lymphatics. Immunohistochemical demonstration of lymphatic vessels in these primary leiomysarcomas was of prognostic significance with regard to the development of lymph node metastasis, and it could be argued that SLNB is indicated in primary leiomyosarcomas of the extremities where intratumoural lymphatics are identified.

We found a high rate of local recurrence in extremity deep soft tissue leiomyosarcoma patients with 25.9% experiencing recurrence despite adequate resection and adjuvant radiotherapy. Mankin and Hornicek report a recurrence rate of 10.8% in 65 patients with leiomyosarcoma [19]. Again this study did not differentiate between leiomyosarcoma of the extremities and other sites. The findings of the present study indicate that deep soft tissue leiomyosarcoma of the extremities, in contrast to leiomyosarcoma arising at other sites has a greater propensity to local recurrence. Such recurrences are difficult to treat and surgical resection of an already irradiated area remains the only option.

## Conclusion

This study has shown that patients with leiomyosarcoma of deep soft tissues of the extremities have a rate of lymph node metastasis of 7% and a local recurrence rate of 25.9% despite adequate excision and post-operative radiotherapy. On the basis of this study, we do not advocate the use of SLNB to this group of patients except in cases where lymphatics can be demonstrated in the primary tumour. Our findings emphasise the importance of close follow up, especially for high grade leiomyosarcomas, particularly in the first year post surgery, as there is a high incidence of local recurrence.

## Competing interests

The authors declare that they have no competing interests.

## Authors' contributions

HG, PC, MG and DW contributed to the design of the study. HG, MG and PC conducted the study. NA carried out pathological studies and MJL, HPG, MG and NA wrote the paper. All authors have read and approved the final manuscript.
